# Effects of Hydrostatic Pressure Treatment of Newly Fertilized Eggs on the Ploidy Level and Karyotype of Pikeperch *Sander lucioperca* (Linnaeus, 1758)

**DOI:** 10.3390/life11121296

**Published:** 2021-11-26

**Authors:** Jenő Káldy, Eszter Patakiné Várkonyi, Georgina Lea Fazekas, Zoltán Nagy, Zsuzsanna J. Sándor, Katalin Bogár, Gyula Kovács, Mariann Molnár, Bence Lázár, Katalin Goda, Zsuzsanna Gyöngy, Zsuzsanna Ritter, Péter Nánási, Ákos Horváth, Uroš Ljubobratović

**Affiliations:** 1 Research Center of Fisheries and Aquaculture, Institute of Aquaculture and Environmental Safety, Hungarian University of Agriculture and Life Sciences, H-2100 Gödöllő, Hungary; Fazekas.Georgina.Lea@uni-mate.hu (G.L.F.); Nagy.Zoltan84@uni-mate.hu (Z.N.); jakabne.sandor.zsuzsanna@uni-mate.hu (Z.J.S.); Bogar.Katalin@uni-mate.hu (K.B.); Kovacs.Gyula@uni-mate.hu (G.K.); Ljubobratovic.Uros@uni-mate.hu (U.L.); 2 National Centre for Biodiversity and Gene Conservation, Institute for Farm Animal Gene Conservation, H-2100 Gödöllő, Hungary; varkonyi.eszter@nbgk.hu (E.P.V.); molnar.mariann@nbgk.hu (M.M.); lazar.bence@nbgk.hu (B.L.); 3 Doctoral School of Animal Biotechnology and Animal Science, Hungarian University of Agriculture and Life Sciences, H-2100 Gödöllő, Hungary; 4 Festetics György Doctoral School, Hungarian University of Agriculture and Life Sciences, H-2100 Gödöllő, Hungary; 5 Animal Biotechnology Department, Institute of Genetics and Biotechnology, Hungarian University of Agriculture and Life Sciences, H-2100 Gödöllő, Hungary; 6 Department of Biophysics and Cell Biology, Faculty of Medicine, University of Debrecen, H-4032 Debrecen, Hungary; goda@med.unideb.hu (K.G.); gyongy.zsuzsanna@med.unideb.hu (Z.G.); ritter.zsuzsanna@med.unideb.hu (Z.R.); peternanasi@med.unideb.hu (P.N.J.); 7 Doctoral School of Molecular Cell and Immune Biology, University of Debrecen, H-4032 Debrecen, Hungary; 8 Department of Aquaculture, Institute of Aquaculture and Environmental Safety, Hungarian University of Agriculture and Life Sciences, H-2100 Gödöllő, Hungary; Horvath.Akos@uni-mate.hu

**Keywords:** triploid, mosaic karyotype, pressure duration, chromosome number, PSI

## Abstract

We studied the effect of different magnitudes (7000 PSI (48.26 MPa), 8000 PSI (55.16 MPa), and 9000 PSI (62.05 MPa)) of hydrostatic pressure on the ploidy of pikeperch larvae. Pressure shock was applied 5 min after the fertilization of eggs at a water temperature of 14.8 ± 1 °C. A 7000 PSI pressure shock was applied for 10 or 20 min, while 8000 and 9000 PSI treatments lasted for 10 min. Each treatment with its respective control was completed in triplicate, where different females’ eggs served as a replicate. In the treatment groups exposed to 7000 PSI for 10 min, only diploid and triploid larvae were identified, while 2n/3n mosaic individuals were found after a 20-min exposure to a 7000 PSI pressure shock. The application of 8000 or 9000 PSI pressure shocks resulted in only triploid and mosaic individuals. Among larvae from eggs treated with 8000 PSI, three mosaic individuals with 2n/3n karyotype were identified (4.0 ± 6.9%), while a single (2.0 ± 3.5%) 1n/3n mosaic individual was found in the 9000 PSI-treated group. To our knowledge, this is the first report that demonstrates the induction of a haplo-triploid karyotype by hydrostatic pressure shock in teleost fish. The dominance of triploid individuals with a reasonable survival rate (36.8 ± 26.1%) after 8000 PSI shock supports the suitability of the hydrostatic pressure treatment of freshly fertilized eggs for triploid induction in pikeperch.

## 1. Introduction

The phenomenon of polyploidization means that an organism possesses three or more chromosome sets. It occurs naturally in many taxa of teleost fishes (e.g., Cypriniformes, Salmoniformes, Perciformes, and Siluriformes), as a result of chromosome multiplication during evolution [1,2]. The ancient and most primitive diploid chromosome number in teleosts is 2n = 48 acrocentric chromosomes [3]. The diploid karyotype of all species in the order Perciformes is 2n = 48. The species of this order can be divided into two further groups: the karyotype of genera *Sander* and *Perca* and the species *Gymnocephalus cernuus* represents a more primitive state, with one pair of metacentric chromosomes, while the species of genera *Zingel* (which have two and three pairs of metacentric chromosomes) and two species of *Gymnocephalus* (*Gymnocephalus baloni* and *Gymnocephalus schraetser,* which have five pairs of metacentric chromosomes) possess a more advanced karyotype [4]. For the genus *Sander*, which includes the species pikeperch (*Sander lucioperca*), naturally occurring polyploid stocks have not yet been described. On the other hand, applying heat shock, cold shock, pressure shock, or cytoskeleton-targeting drugs (cytochalasin B or colchicine) induces polar body retention in fishes, resulting in polyploid individuals [5]. When fertilized eggs were treated with pressure, cold, or heat shock shortly after fertilization, triploid progenies were obtained, because the treatment inhibits the expulsion of the second polar body from the egg during the second meiotic division [6,7,8]. In this case, the triploid offspring inherits two maternal and one paternal chromosome sets [9]. The pressure shock can cause chromosomal retention of maternal or paternal origin. Glover et al. (2020) [10] showed that the application of pressure shock in the Atlantic salmon (*Salmo salar*) species can lead to various chromosomal aberrations. Chromosomal aberrations were identified in 0.9% of triploid individuals, and mosaic individuals were also found among the non-hatched embryos. In the case of a tetraploid walleye produced using a pressure shock, aneuploid and mosaic individuals were also found at different rates, ranging from 0 to 17% [11]. In most cases, DNA staining combined with flow cytometry was applied to determine the ploidy level of individuals and the effectiveness of treatments. However, in some cases, the flow cytometer identified diploid individuals that were hyperdiploid aneuploid individuals [12]. Therefore, besides flow cytometry-based DNA content analysis, chromosome preparation techniques [13], as well as microsatellite marker analyses, are used to accurately validate various chromosomal abnormalities [10].

The production of triploids can be important for aquaculture for several reasons. On the one hand, induced triploid fish are either partially or completely sterile, and the reduction of gonadal development can result in improved growth performance [14]. In addition, the faster growth and higher filet yield of triploids can result in higher profits [15]. The optimal conditions for the growth of diploids and triploids are often considered to be equal, yet, it is often observed that triploids show a reduced growth rate compared to diploids in their early life stages, while, later on, they grow at a similar or better rate [16]. On the other hand, artificially produced triploids of several species have successfully been used in surrogate gamete production methods as recipients, including salmonids [17], medaka [18], and grass buffer [19]. Triploid zebrafish (*Danio rerio*) have successfully been used for intraperitoneal germ cell transplantation [20]. This method, combined with cryopreservation of spermatogonial stem cells, could be an alternative approach for conserving valuable and endangered genetic resources [21]. The application of this protocol could be very important for intensive pikeperch aquaculture.

In the present study, we conducted a series of trials to determine the effect of pressure shocks of different magnitudes and duration times on the ploidy level of pikeperch, with the final aim of developing a reliable protocol for triploidy induction in this species.

## 2. Materials and Methods

### 2.1. Hydrostatic Pressure Shock Treatment

Altogether, 12 female pikeperch individuals were used in this study, where eggs of each individual were used for one replicate and its respective control. Artificial reproduction was performed as explained by Ljubobratović et al. (2021) [22]. A total of 100 g of eggs per female were used for each replicate and were separated (treatment and control) after the fertilization and egg adhesiveness elimination treatment and about 1 min prior to pressure shock. The uniquely produced ‘Hydra’ pressure chamber (Hydra Technology Service Ltd., Budapest, Pest, Hungary) was 1–5 liters in volume and had a 700 Bar maximum performance. During the experiment, three different magnitudes of pressure treatment were used in triplicate: 7000 PSI (48.26 MPa), 8000 PSI (55.16 MPa), and 9000 PSI (62.05 MPa). In the case of 7000 PSI, two different duration times (10 and 20 min) were tested. The time of initiation (TI) for the pressure shock was 5 min after fertilization in each case. Fertilization took place in 0.5 L of clean fertilization water at 14.8 ± 0.1 °C for 1 min, and then 0.5 L of freshwater mixed with 1 mL alkalase enzyme solution was added, to remove the adhesiveness of the eggs [23]. In this 1 mL L-1 alkalase solution, the eggs were mixed for 2 min, and then the eggs were washed twice with fertilizing water. Later, the eggs were divided into equal portions: control and treated (pressurized) replicate. After the respective durations of pressure shock, the pressure was released with the control valve in less than one second. The eggs were then mixed in clean fertilizing water for an additional 20 min and then placed in separate Zuger jars. During incubation, the water temperature was kept at 14.6 ± 1 Co. The embryo survival rate (ESR) was calculated after 72 h, as explained by Ljubobratović et al. (2019) [24]:

Embryo survival = (volume of eggs 72 h after fertilization)/(volume of eggs at the time of stocking in Zuger jar) × mean percentage of live eggs

Eggs hatched after about 8 days of incubation, when each group of larvae was transported into separate 20-L aquariums for further samplings. The karyotype of the larvae was determined before the onset of exogenous feeding by applying chromosome analysis and laser scanning cytometry (LSC).

### 2.2. Chromosome Analysis of Pikeperch Larvae from the Treated and Control Groups

A direct method was used for chromosome preparation from 5–8 days post-hatch (DPH) pikeperch larvae. Larvae were pre-treated with a mitostatic agent (0.05% KaryoMAX™ Colcemid™ Solution: Thermo Fisher Scientific, (Waltham, MA, USA)) for 3–3.5 h in well-aerated freshwater, and then they were put into hypotonic solution (distilled water) for 25 to 35 min. Subsequently, the hypotonic solution was replaced by fresh fixative (methanol/acetic acid applied at a volume ratio of 3:1) for 20 min. The fixative was changed three times. Larvae were stored in fixative at 4 °C, until the preparation of slides.

Fixed larvae were put into a Petri dish for drying (1–2 min). Cells were dispersed with a micropipette in a few drops of 50% acetic acid and were then dropped onto a microscope slide, pre-warmed on a 50 °C heating plate (BIOSAN Thermo Block TDB-120), and then removed very slowly using a pipette. This step was repeated several times (modified after Várkonyi et al., 1998 [17]). The slides were dried and stained with 5% of KaryoMAX™ (Waltham, MA, USA) Giemsa Stain Solution in phosphate buffer pH 7.0 (Gibco™ 10092013, Waltham, MA, USA) for 7–8 min. The samples for chromosome analysis were taken separately from each larva. A total of 30 larvae were analyzed from each treatment and control group. Two slides were prepared from each individual, and at least 30 metaphase spreads per individual were examined, to analyze the chromosome number.

### 2.3. Sample Preparation for Laser-Scanning Cytometry from the Hydrostatic-Pressure-Treated and Control Groups

For laser-scanning cytometry analysis, we prepared single-cell suspensions from 6–8 DPH pikeperch larvae. Each larva was incubated in 50 µL of proteinase K solution (0.5 mg/mL) in phosphate-buffered saline (PBS, pH = 7.4) containing 1 mM EGTA for 10 min at 37 °C and then dissected using harsh pipetting. After washing with PBS, the samples were centrifuged at 300× *g* for 5 min at 4 °C and then fixed and permeabilized by 70% ethanol for 30 min at room temperature. Thereafter, the samples were washed with PBS (500× *g* for 5 min at 4 °C) and transferred to 96-well plates. Before LSC measurement, the samples were stained with 50 µg/mL propidium iodide (PI) for 20 min at room temperature and washed again with PBS (500× *g* for 5 min at room temperature).

### 2.4. Laser-Scanning Cytometry Measurements and Analysis of Data

LSC imaging was performed using an iCys instrument (iCys^®^ Research Imaging Cytometer; CompuCyte, (Westwood, MA, USA)). PI was excited using a 488 nm Argon ion laser, and its fluorescence signal was collected through a 650 nm long-pass filter using a UPlan FI 20× (NA 0.5) objective. Each viewing field (comprising 1000 × 768 pixels) was scanned with a step size of 1.5 μm. Data evaluation was performed using iCys 7.0 software for Windows XP. G1 phase cells were selected in scatterplots based on the nuclear area and PI fluorescence intensity of cells. The PI fluorescence intensity integral values (i.e., the summed fluorescence intensity of all the pixels representing the nucleus of a cell) were measured for all cells and averaged by LSC. The median PI fluorescence integrals (±SD) were determined for about 500–1000 G1 phase cells per larva using iCys software.

### 2.5. Statistical Analysis

All data were analyzed using R Studio (v1.4.1106). R software was used to build a logistic regression model (generalized linear model) in which ‘embryo survival’ was the response variable, while ‘treatment type’ was the predictor variable. The predictor variable showed a significant effect on the ratio of surviving embryos; therefore, multiple comparisons of means (Tukey contrasts) were performed to further analyze the differences between groups. In case of the karyotype data, R software was used to perform Fisher’s exact tests, to compare the ratios of the three genotypes between the treated group pairs. (* *p* < 0.05, ** *p* < 0.01, *** *p* < 0.001.)

## 3. Results

### 3.1. Chromosome Analysis

**Figure 1:**
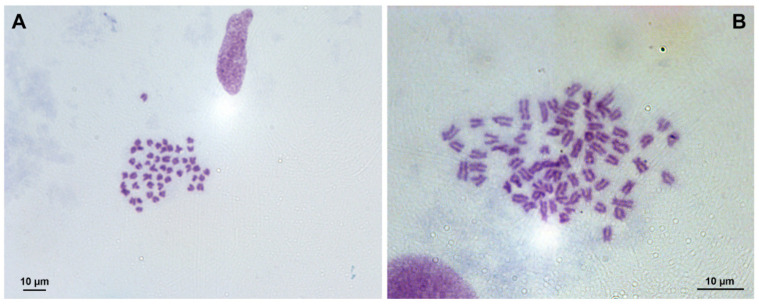
Mitotic metaphase chromosome spread (**A**) from a control diploid larva (2n = 48) and a pressure shock treated (**B**) triploid larva (3n = 72) of pikeperch. Bar is 10 µm.

Direct preparations (Figure 1) for chromosome analysis were prepared from 30 larvae from each experimental (1T, 2T, 3T, 4T, 6T, 7T, 8T 9T 10T 11T, and 12T) and control (1K, 2K, 3K, 4K, 6K, 7K, 8K 9K 10K 11K, and 12K) group.

Due to technical issues, appropriate samples for evaluation could not be obtained from every larva. Therefore, a total of 371 larval chromosome numbers were determined, as shown in Table 1. From the three replicates of the first treatment (710Ta, 710Tb, and 710Tc), altogether, 32 individuals could be analyzed, and in the corresponding controls (710Ca, 710Cb, and 710Cc), 42 larvae suitable for analysis were obtained. The chromosome number was determined from 55 individuals in the three treated replicates of the second treatment (720Ta, 720Tb, and 720Tc) and 50 individuals in the corresponding controls (720Ca, 720Cb, and 720Cc). Chromosome numbers were determined from 58 individuals in the three independent replicates of the third treatment (810Ta, 810Tb, and 810Tc) and 61 individuals from the corresponding controls (810Ca, 810Cb, and 810Cc). There were no live larvae in one of the three treated replicates of the fourth treatment; therefore, the chromosome number could be determined from the 32 individuals in the remaining two groups (910Ta, 910Tb), and 41 from the corresponding controls (910Ca and 910Cb) were analyzed.

**Figure 2:**
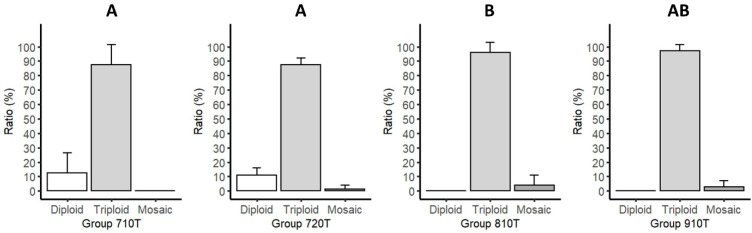
Comparison of the ratio of diploid, triploid, and mosaic pikeperch larvae hatched from eggs treated with 7000 PSI for 10 (Group 710T) or 20 min (Group 720T), or with 8000 PSI (Group 810T) or 9000 PSI (Group 910T) for 10 min, following fertilization to induce triploidy. Fisher’s exact tests were used to compare the ratios of the three karyotypes between the groups. A significant difference was found between groups 710T-810T and 720T-810T. Different letters indicate significant differences between groups (A and B), (*p* < 0.05 was considered significant). Abbreviations: 710T: treated groups with 7000 PSI pressure and 10 min duration time; 720T: treated groups with 7000 PSI pressure and 20 min duration time; 810T: treated groups with 8000 PSI pressure and 10 min duration time; 910T: treated groups with 9000 PSI pressure and 10 min duration time.

In terms of karyotype ratio, the 810T group was significantly different from the 710T and 720T groups. In the case of the other comparisons, there were no significant differences in karyotype ratio between the groups (Figure 2).

**Figure 3:**
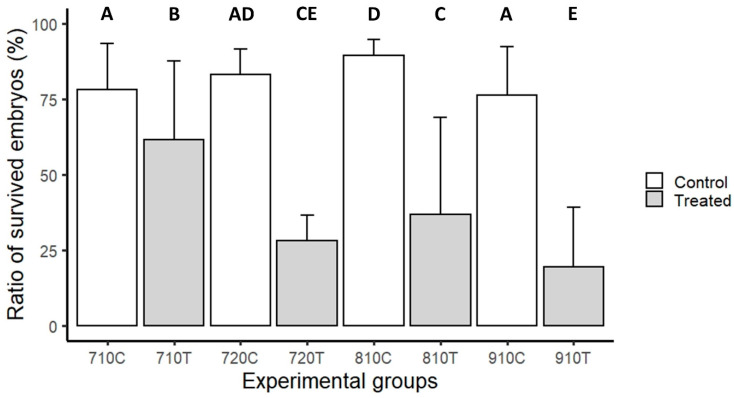
Comparison of the survival rates of pikeperch larvae hatched from eggs treated with 7000 PSI for 10 (710T) or 20 min (720T), or with 8000 PSI (810T) or 9000 PSI (910T) for 10 min following fertilization to induce triploidy and their controls (710C, 720C, 810C, and 910C). Different letters indicate significant differences between groups (A, B, C, D and E). After fitting a logistic regression model, multiple comparisons of means (Tukey contrasts) were performed to analyze the differences between groups. (*p* < 0.05 was considered significant). Abbreviations: 710C: control groups of 710T-treated groups; 710T: treated groups with 7000 PSI pressure and 10 min duration time; 720C: control groups of 720T-treated groups; 720T: treated groups with 7000 PSI pressure and 20 min duration time; 810C: control groups of 810T-treated groups; 810T: treated groups with 8000 PSI pressure and 10 min duration time; 910C: control groups of 910T-treated groups; and 910T: treated groups with 9000 PSI pressure and 10 min duration time.

The embryo survival rate was significantly lower in all four hydrostatic-pressure-treated groups compared to their respective controls. Among the treated groups, the survival rate was significantly higher in the 710T group compared to all other treated groups. The group 720T showed no significant differences compared to the 810T and 910T groups; while, the 810T group had a significantly higher survival rate than the 910T group (Figure 3).

### 3.2. Laser-Scanning Cytometry Measurement

**Figure 4:**
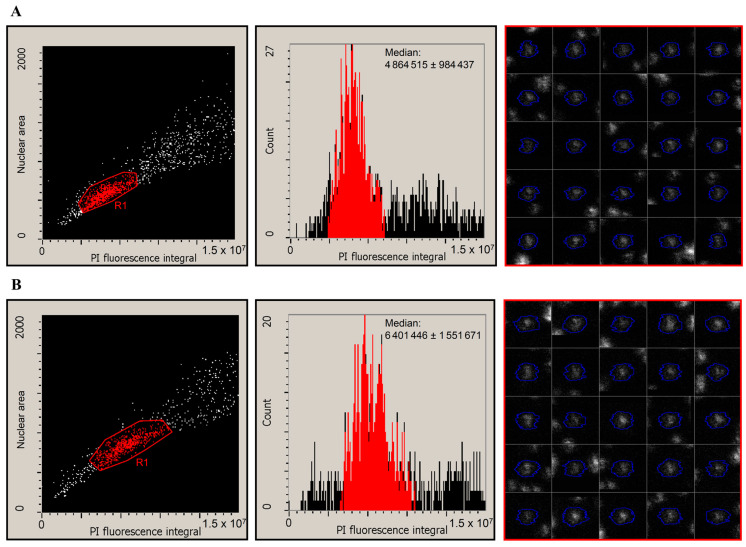
Laser-scanning cytometry (LSC) measurement of the nuclear size and DNA content of propidium-iodide (PI)-stained pikeperch larvae. Panels (**A**,**B**) show scatterplots (left panels) and DNA distribution histograms (middle panels) of cells from a representative larva from the 710Cb untreated diploid control group and a triploid larva from the 710Tb group treated with a 7000 PSI pressure for 10 min, respectively. G1 phase nuclei are marked by the R1 gate in the scatterplot and are indicated by red color in the histogram. Examples of G1 phase nuclei are encircled by a blue color in the galleries (right panels).

Pikeperch larvae are of a rather small body size before the onset of first feeding. We could isolate a relatively small number of cells from the embryos, which were difficult to analyze using flow cytometry. Therefore, in a pilot experiment, we applied the LSC technique to determine the ploidy level of pikeperch larvae using 1000–5000 cells prepared from one individual (Figure 4). Based on the nuclear area and PI fluorescence intensity, we could discriminate intact G0/G1 phase cells from cell debris and S/G2/M phase cells (Figure 4, left panels). We determined the median PI fluorescence intensity of the G1 peak from larvae belonging to the diploid control group (for example, see Figure 4A, middle panel) and the 7000 PSI-pressure-treated group (Figure 4B, middle panel).

## 4. Discussion

Our experiments demonstrated that 7000 PSI pressure shocks resulted in an approximately 90% triploid ratio in pikeperch. Previous studies carried out on species from the Percidae family demonstrated a lower triploid ratio compared to our results [8,11]. However, they observed that the triploid ratio improved when increasing the length of the pressure shock, from 3–6 min to 8–12 min [11]. In pikeperch, by increasing the duration of the treatment from 10 min to 20 min, we did not find a significant difference in the triploid ratio; while, the embryo survival rate significantly decreased. In the group treated with 7000 PSI for 20 min, we detected one mosaic larva with a 2n/3n mosaic karyotype. In the case of heat shock, 2n/3n mosaic individuals were found in less than 1% of yellow perch (*Perca flavescens*) individuals if the onset of the shock began 5 min after fertilization [25]. In some cases, shock-induced polyploidization may result in non-uniform polyploid stocks, but in part genetically mosaic stocks. Lemoine et al. (1980) [26] also obtained polyploid mosaic specimens from the larvae of cold-shock-treated brook trout (*Salvelinus fontinalis*) eggs. According to that study, a possible explanation for the mosaicism is that the second polar body is retained but does not fuse with the sperm, resulting in binuclear cells. Polyploid–diploid mosaic individuals were also reported by Smith and Lemoine (1979) [27] in the case of larvae (fry) hatched from colchicine-treated brook trout eggs and in sturgeon fishes treated by heat shock [28]. Allen and Stanley (1979) [29] found triploid–diploid mosaic individuals among the larvae of Atlantic salmon, when fertilized eggs were treated with cytochalasin B. Refstie et al. (1977) [30] found tetraploid–diploid mosaic larvae when salmon and rainbow trout eggs were treated with cytochalasin B after fertilization. This was explained by the non-disjunction of each cell. Our study, for the first time, shows this phenomenon in pikeperch eggs treated with hydrostatics pressure.

In the case of pikeperch eggs treated with a 8000 PSI pressure, we identified 5.2 ± 2.4% mosaic larvae with 2n/3n karyotype. The triploid ratio was significantly higher than in the groups treated with 7000 PSI, either for 10 or 20 min. Nevertheless, the survival rate for larvae also decreased at higher pressure. No diploid individuals were found in the 8000 PSI treatment groups; therefore, only the presence of mosaic larvae was responsible for the triploid ratio differing from 100%. In accordance with our experiments, Malison et al. (2001) [11] also achieved a higher triploid ratio in their 8000 PSI-treated group compared to 7000 PSI. Nevertheless, in that study, half an hour was the shortest reported pressure shock duration that could result in the full elimination of diploids.

The triploid ratio of the 9000 PSI-treated group did not differ significantly from the 8000 PSI-treated group, while the survival rate was significantly lower. This is in contrast with the results of studies in walleye, where the triploid ratio increased with higher pressure [11]. However, a comparable treatment of 12 min duration yielded a survival rate of about 80.0% in walleye [8], while it was below 20% in the present study with pikeperch. Fethermann et al. (2015) [31] found that in walleye at 9500 PSI, the maximum triploid ratio was reached after 10 min of treatment, when the time from fertilization to the onset of shock was 7 min 33 s. The maximum triploid ratio obtained in the walleye was 98.5% [31]. These results agree with the values we obtained for pikeperch at 8000 and 9000 PSI. Generally, in percids, the application of cold shock [32], heat shock [14,25,33,34], or pressure shock [11,35] resulted in a 100% triploid ratio, but with rather variable survival rates. Reviewing the previously mentioned results and the results of the present study, it appears that pressure shock is an effective tool for inducing triploidy in percids; however, special attention should be paid to finding the minimal effective exposure duration and magnitude, to improve embryo survival rates.

A mosaic individual with a 1n/3n karyotype was also detected in 3.13 ± 1.5% of this treatment group. Haplodiploid mosaic individuals have already been found in several fish species as a result of various shock effects following fertilization, as well as being a consequence of polyspermic fertilization. Swarup (1959) [36] found haplodiploid genetic mosaicism in three-spined stickleback (*Gasterosteus aculeatus*) using cold shock. This was explained by the fact that the sperm cell did not fuse with the female pronucleus, resulting in the formation of a diploid and a haploid cell line. Svardson (1945) [37] also found haplodiploid mosaicism in whitefish (*Coregonus lavaretus*) larvae hatched from cold-shock-treated eggs. According to his explanation, the haploid line may come from a non-rejected polar body, which has begun to develop. Iegorova et al. (2018) [38] reported that the interspecific polyspermy fertilization between odd ploidy sturgeon parent species caused viable haplodiploid progeny.

However, the interspecific polyspermic fertilization between odd ploidy levels of sturgeon parent species by the retention of a second polar body can also result in 1n/3n abnormally divided mosaic individuals [39].

In amphibians, 0–11% of fertilized eggs were found to develop into haplo-triploid mosaic individuals, following exposure to pressure shock. The ratio of these haplo-triploid individuals increased in parallel with the pressure elevation, from 3000 to 7000 PSI [40]. That study did not identify diploid individuals in the treatment groups exposed to pressure shock. Mosaic individuals have also been described upon heat-induced triploidization in less than 1% of walleye larvae [25]. In that species, heat-induced tetraploidization was also reported [8]. However, the karyotype of these mosaics was not published. In amphibians, it was previously found that pressure shock can inhibit the rejection of the second polar body, leading to the development of haplo-triploid mosaic individuals from the fertilized eggs [40]. The haploid cell line was of androgenic origin, and its occurrence was not time-bounded [40]. To the best of our knowledge, this study is the first observation of pressure-shock-induced haplo-triploid mosaicism in the teleost fishes.

## 5. Conclusions

Our experiments demonstrated that a hydrostatic pressure shock applied 5 min after fertilization effectively induces triploidy in pikeperch. However, in response to both 10 and 20 min of 7000 PSI pressure shock, diploid individuals were still present among the hatched larvae. On the other hand, in both 8000 and 9000 PSI treatments, diploid larvae were not found. Both a prolonged treatment time and increased pressure reduced the embryo survival rate and induced the formation of mosaic individuals, although at a low percentage. Either a 20 min exposure to the 7000 PSI pressure shock or 10 min exposure to the 8000 PSI pressure yielded some 2n/3n mosaic individuals, while a 10 min 9000 PSI pressure shock induced 1n/3n mosaicism at a low level. Taken together, our results support that in pikeperch, a 10 min pressure shock of 8000 PSI is appropriate for inducing the triploid karyotype, with an acceptable embryo survival rate; although a low number of mosaic individuals also appeared. Automated microscopy techniques, such as LSC, can be applied for DNA content analysis and ploidy determination when there is an insufficient number of cells for flow cytometry evaluation.

## Figures and Tables

**Table 1:** Effect of four different pressure shock treatments on the genome size of pikeperch larvae, based on chromosome analysis. All treatments were performed in triplicate with parallel control studies.

Pressure (PSI)	Duration (min)	Type	Replicate	Identifier	Diploid Fish (pcs)	Triploid Fish (pcs)	Mosaic Fish (pcs)	Karyotyped Fish (pcs)	Diploid (%)/ Triploid (%)
-	-	Control	1	710C_a_	11	0	0	11	
			2	710C_b_	15	0	0	15	100.0 ^a^/0.0 ^b^
			3	710C_c_	16	0	0	16	
7000	10	Treated	1	710T_a_	1	9	0	10	12.3 ± 11.1 ^c^/ 87.7 ± 11.1 ^d^
			2	710T_b_	3	8	0	11	
			3	710T_c_	0	11	0	11	
-	-	Control	1	720C_a_	15	0	0	15	
			2	720C_b_	19	0	0	19	100.0 ^a^/0.0 ^b^
			3	720C_c_	16	0	0	16	
7000	20	Treated	1	720T_a_	2	24	0	26	11.3 ± 4.0 ^c^/ 87.0 ± 3.7 ^d^
			2	720T_b_	1	5	0	6	
			3	720T_c_	2	20	1	23	
-	-	Control	1	810C_a_	27	0	0	27	
			2	810C_b_	21	0	0	21	100.0 ^a^/0.0 ^b^
			3	810C_c_	13	0	0	13	
8000	10	Treated	1	810T_a_	0	22	3	25	0.0 ^e^/ 96.0 ± 5.7 ^f^
			2	810T_b_	0	14	0	14	
			3	810T_c_	0	19	0	19	
-	-	Control	1	910C_a_	15	0	0	15	
			2	910C_b_	26	0	0	26	100.0 ^a^/0.0 ^b^
			3	910C_c_	17	0	0	17	
9000	10	Treated	1	910T_a_	0	15	0	15	0.0 ^e^/ 97.0 ± 3.0 ^df^
			2	910T_b_	0	16	1	17	
			3	910T_c_	0	0	0	0	

Abbreviations: PSI: pound per square inch, min: minute; pcs: pieces; a–f in superscript: significant difference; 710C_a-b-c_: control groups of 710T_a-b-c_ in three replicates; 710T_a-b-c_: treated groups with 7000 PSI pressure and 10 min duration time; 720C_a-b-c_: control groups of 720T_a-b-c_ in three replicates; 720T_a-b-c_: treated groups with 7000 PSI pressure and 20 min duration time; 810C_a-b-c_: control groups of 810T_a-b-c_ in three replicates; 810T_a-b-c_: treated groups with 8000 PSI pressure and 10 min duration time; 910C_a-b-c_: control groups of 910T_a-b-c_ in three replicates; 910T_a-b-c_: treated groups with 9000 PSI pressure and 10 min duration time.

## Data Availability

Not applicable.

## References

[1] Tóth B., Várkonyi E., Hidas A., Edviné Meleg E., Váradi L. (2005). Genetic analysis of offspring from intra- and interspecific crosses of Carassius auratus gibelio by chromosome and RAPD analysis. J. Fish Biol..

[2] Zhou L., Gui J. (2017). Natural and artificial polyploids in aquaculture. Aquac. Fish..

[3] Ohno S. (1970). Evolution by Gene Duplication.

[4] Ráb P., Roth P., Mayr B. (1987). Karyotype Study of Eight Species of European Percid Fishes (*Pisces, Percidae*). Caryologia.

[5] Ihssen P.E., McKay L.R., McMillan I., Phillips R.B. (1990). Ploidy Manipulation and Gynogenesis in Fishes: Cytogenetic and Fisheries Applications. Trans. Am. Fish. Soc..

[6] Rougeot C. (2015). Sex and Ploidy Manipulation in Percid Fishes. Biology and Culture of Percid Fishes.

[7] Chourrout D. (1984). Pressure-induced retention of second polar body and suppression of first cleavage in rainbow trout: Production of all-triploids, all-tetraploids, and heterozygous and homozygous diploid gynogenetics. Aquaculture.

[8] Malison J.A., Terrence B.K., James A.H., Terence P.B., Clyde H.A. (1993). Manipulation of ploidy in yellow perch (*Perca flavescens*) by heat shock, hydrostatic pressure shock, and spermatozoa inactivation. Aquaculture.

[9] Yamazakia F., Goodierb J. (1993). Cytogenetic effects of hydrostatic pressure treatment to suppress the first cleavage of salmon embryos. Aquaculture.

[10] Glover K.A., Harvey A.C., Hansen T.J., Fjelldal P.G., Besnier F.N., Bos J.B., Ayllon F., Taggart J.B., Solberg M.F. (2020). Chromosome aberrations in pressure induced triploid *Atlantic salmon*. BMC Gen..

[11] Malison J.A., Held J.A., Weil L.S., Terrence B.K., Thorgaard G.H. (2001). Manipulation of Ploidy in Walleyes by Heat Shock and Hydrostatic Pressure Shock. N. Am. J. Aquac..

[12] Devlin R.H., Sakhrani D., Biagi C.A., Ki-Whan E. (2010). Occurrence of incomplete paternal-chromosome retention in GH-transgenic coho salmon being assessed for reproductive containment by pressure-shock-induced triploidy. Aquaculture.

[13] Várkonyi E., Bercsényi M., Ozouf-Costaz C., Billard R. (1998). Chromosomal and morphological abnormalities caused by oocyte ageing in *Silurus glanis*. J. Fish Biol..

[14] Rougeot C., Minet L., Prignon C., Vanderplasschen A., Detry B., Pastoret P.-P., Mélard C. (2003). Induce triploidy by heat shock in *Eurasian perch*, *Perca fluviatilis*. Aquat. Living Resour..

[15] Piferrer F., Beaumont A., Falguière J.C., Flajšhans M., Haffray P., Colombo L. (2009). Polyploidfish and shellfish: Production, biology and applications to aquaculture for performance improvement and genetic containment. Aquaculture.

[16] Fraser W.K.T., Fjelldal P.G., Hansen T., Mayer I. (2012). Welfare Considerations of Triploid Fish. Rev. Fish. Sci..

[17] Lee S., Katayama N., Yoshizaki G. (2016). Generation of juvenile rainbow trout derived from cryopreserved whole ovaries by intraperitoneal transplantation of ovarian germ cells. Biochem. Biophys. Res. Commun..

[18] Seki S., Kusano K., Lee S., Iwasaki Y., Yagisawa M., Ishida M., Hiratsuka T., Sasado T., Naruse K., Yoshizaki G. (2017). Production of the medaka derived from vitrified whole testes by germ cell transplantation. Sci. Rep..

[19] Hamasaki M., Takeuchi Y., Yazawa R., Yoshikawa S., Kadomura K., Yamada T., Miyaki K., Kikuchi K., Yoshizaki G. (2017). Production of tiger puffer *Takifugu rubripes* offspring from triploid grass puffer *Takifugu niphobles* parents. Mar. Biotechnol..

[20] Franĕk R., Tichopád T., Fučíková M., Steinbach C., Pšenička M. (2019). Production and use of triploid zebrafish for surrogate reproduction. Theriogenology.

[21] Marinovic Z., Lujic J., Kasa E., Csenki Z., Urbanyi B., Horvath A. (2018). Cryopreservation of zebrafish spermatogonia by whole testes needle immersed ultra-rapid cooling. J. Vis. Exp..

[22] Ljubobratovič U., Kwiatkowski M., Tóth F., Demény F. (2021). Effects of hormonal treatment before water warming on synchronisation of spawning time, oocyte size, and egg quality in pikeperch (*Sander lucioperca*). Anim. Reprod. Sci..

[23] Kristan J., Blecha M., Policar T. (2015). Alcalase treatment for elimination of stickiness in pikeperch (*Sander lucioperca* L.) eggs under controlled conditions. Aquac. Res..

[24] (2019). Uroš Ljubobratović, Géza Péter, Rene Alvestad, Zoltán Horváth, András Rónyai: Alcalase enzyme treatment affects egg incubation and larval quality in *pikeperch* (*Sander lucioperca*). Aquac. Int..

[25] Malison J.A., Procarione L.S., Held J.A., Kayes T.B., Amundson C.H. (1993). The influence of triploidy and heat and hydrostatic pressure shocks on the growth and reproductive development of juvenile yellow perch (*Perca flavescens*). Aquaculture.

[26] Lemoine H.L., Smith T.L. (1980). Polyploidy Induced in Brook Trout by Cold Shock. Trans. Am. Fish. Soc..

[27] Smith L.T., Lemoine H.L. (1979). (1979) Colchicine-Induced Polyploidy in Brook Trout. Progress. Fish-Cult..

[28] Lebeda I., Ráb P., Majtánová Z., Flajšhans M. (2020). Artifcial whole genome duplication in paleopolyploid sturgeons yields highest documented chromosome number in vertebrates. Sci. Rep..

[29] Allen S.K., Stanley J.G. (1979). Polyploid Mosaics Induced by Cytochalasin B in Landlocked Atlantic Salmon Salmo salar. Trans. Am. Fish. Soc..

[30] Refstie T., Vassvik V., Gjedrem T. (1977). Induction of polyploidy in salmonids by cytochalasin B. Aquaculture.

[31] Fetherman E.R., Lepak J.M., Brown B.L., Harris D.J. (2015). Optimizing Time of Initiation for Triploid Walleye Production Using Pressure Shock Treatment. North Am. J. Aquac..

[32] Dadras H., Blecha M., Malinovskyi O., Flajšhans M., Lebeda I., Křišťan J., Policar T. (2021). Triploidization in pikeperch (*Sander lucioperca*) induced by cold shock. Aquaculture.

[33] Garcia-Abiado M.A.R., Lynch W.E., Dabrowski K., Hartman T. (2001). Use of Thermal and Pressure Shocks to Induce Triploid Hybrid Saugeyes. North Am. J. Aquac..

[34] Blecha M., Flajshans M., Lebeda I., Kristan J., Svacina P., Policar T. (2016). Triploidisation of pikeperch (*Sander lucioperca*), first success. Aquaculture.

[35] Garcia-Abiado M.A., Penn M., Dabrowski K., Stafford J. (2007). Evaluation of Two Commercially Available Pressure Chambers to Induce Triploidy in Saugeyes. N. Am. J. Aquac..

[36] Swarup H. (1959). Production of triploidy ingasterosteus *aculeatus* (L). J. Genet..

[37] Svardson G. (1945). Chromosome Studies on *salmonidae*. Ph.D. Thesis.

[38] Iegorova V., Psenicka M., Lebeda I., Rodina M., Saito T. (2018). Polyspermy produces viable haploid/diploid mosaics in sturgeon. Biol. Rep..

[39] Iegorova V., Havelka M., Psenicka M., Saito T. (2018). First evidence of viable progeny from three interspecific parents in sturgeon. Fish Phys. Biochem..

[40] Briedis A., Elinson R. (1982). Suppression of Male Pronuclear Movement in Frog Eggs by Hydrostatic Pressure and Deuterium Oxide Yields Androgenetic Haploids. J. Exp. Zool..

